# From classroom environment to mathematics achievement: The mediating role of self-perceived ability and subject interest

**DOI:** 10.1016/j.lindif.2016.07.009

**Published:** 2016-08

**Authors:** Maria G. Tosto, Kathryn Asbury, Michèle M.M. Mazzocco, Stephen A. Petrill, Yulia Kovas

**Affiliations:** a Psychology in Education Research Centre, Department of Education, University of York, York YO10 5DD, United Kingdom; b Department of Psychology, Goldsmiths, University of London, London SE14 6NW, United Kingdom; c Department of Psychology, Tomsk State University, 36, Lenina Avenue, Tomsk 634050, Russia; d SGDP Centre (PO80), Institute of Psychiatry, King's College London, De Crespigny Park, London SE5 8AF, United Kingdom; e Institute of Child Development, University of Minnesota, Minneapolis, MN 55455, United States; f Department of Psychology, The Ohio State University, Columbus, OH 43210, United States

**Keywords:** Mathematics, Self-perceived abilities, School achievement, Classroom environment, Interest

## Abstract

Drawing on Bandura's triadic reciprocal causation model, perceived classroom environment and three intrapersonal factors (mathematics self-efficacy, maths interest and academic self-concept) were considered as predictors of test performance in two correlated mathematics assessments: a public examination (GCSE) and an on-line test, both taken by UK pupils at age 16 (n = 6689). Intrapersonal factors were significantly associated with both test scores, even when the alternative score was taken into account. Classroom environment did not correlate with mathematics achievement once intrapersonal factors and alternative test performance were included in the model, but was associated with subject interest and academic self-concept. Perceptions of classroom environment may exercise an indirect influence on achievement by boosting interest and self-concept. In turn, these intrapersonal factors have direct relationships with achievement and were found to mediate the relationship between perceived classroom environment and maths performance. Findings and their implications for mathematics education are discussed.

## Highlights

• Can learning environment (classroom) and intrapersonal factors (self-perceived ability and interest) predict maths at age 16?
• Intrapersonal factors are associated with maths performance at 16.
• Learning environments are associated with intrapersonal factors at this age.
• In particular, perceived maths classroom environment predicts interest in maths.
• Intrapersonal factors mediate the relationship between perceived classroom environment and maths performance.

## 1 Introduction

### 1.1 The importance of mathematics

Maths matters. International surveys predict an increase of almost 1% in annual GDP growth per capita with half a standard deviation's increase in individual maths and science performance (OECD, 2010). In addition to predicting national wealth, mathematical skills are associated with socio-economic well-being. For example, longitudinal research in the UK suggests that people with poor mathematical skills are more than twice as likely as those with better skills to be represented at the lowest level of employment, and are at increased risk of poor mental and physical health (Bynner & Parsons, 2005).

In England and Wales the public examination taken at age 16 (GCSE: General Certificate of Secondary Education) really matters, having life-long implications. GCSE maths is graded from A* (A-star) to G, and Grade C is the minimum requirement for many educational and employment opportunities. Students who do not achieve a C in maths are not eligible to study certain A Level subjects (Advanced qualifications for UK 16 + year olds); will not be accepted by some technical and vocational courses; and are unlikely to be accepted at University. The number of employment opportunities requiring a minimum of Grade C is constantly growing. Grade C, therefore, is a minimum requirement for accessing many opportunities the adult world has to offer, and achieving it is an important hurdle for young people to overcome. And yet, in summer 2014, 42.4% of candidates for GCSE maths achieved less than Grade C (Joint Council for Qualifications, 2014).

### 1.2 Explaining individual differences in mathematics

To some extent, people differ in mathematics achievement because they vary in abilities that are important for learning mathematics. For example, individual differences in maths performance have been found to be associated with individual differences in memory (Swanson & Sachse-Lee, 2001), processing speed (Geary, 2011, Taub et al., 2008), intelligence (Deary, Strand, Smith, & Fernandes, 2007), attention (Dulaney, Vasilyeva, & O'Dwyer, 2015), language ability (Vukovic & Lesaux, 2013) and spatial skills (Rohde and Thompson, 2007, Tosto et al., 2014).

A robust body of research further suggests that individual differences in motivation are also associated with maths achievement (Elliot and Dweck, 2005, Robbins et al., 2004). Academic motivation includes the value that individuals place on a subject, their expectation of succeeding in it (Eccles & Wigfield, 2002), and whether they like and are interested in it (Marsh, 1986). In addition, the place where learning happens has been found to influence learning outcomes (Pianta et al., 2007, Young, 2005). In summary, learning mathematics appears to be linked to factors related to both motivation (intrapersonal factors) and the wider world (learning environments) as consistently as it is with cognitive abilities. Bandura's triadic reciprocal causation model predicts dynamic relationships between intrapersonal factors, behaviour (learning) and the environment (Bandura, 1986, Bandura, 2012) and this theoretical framework forms the basis for the current study's hypotheses. We evaluate the extent to which individual differences in mathematics achievement are associated with three intrapersonal factors and one learning environment.

### 1.3 Self-perceived abilities and interest in maths

The current study assesses self-efficacy for mathematics, academic self-concept and subject interest in relation to mathematics performance and perceptions of the classroom environment.

*Maths self-efficacy* refers to an individual's belief in their ability to perform a specific maths task in a specific context (Bandura, 1977, Pajares, 1996). Self-efficacy beliefs about mathematics tasks have been found to be strongly associated with mathematics achievement (e.g. Hackett and Betz, 1989, Hoffman and Schraw, 2009, Pietsch et al., 2003). The relationship is likely to be reciprocal, as predicted by social cognitive theory: achieving well in maths may foster the belief that you are good at maths which in turn may foster mathematical achievement – a two-way street (Marsh and Craven, 2006, Williams and Williams, 2010).

*Academic self-concept* reflects an individual's assessment of their own general academic self-worth, based on past performance as well as their performance relative to others (Williams & Williams, 2010). Academic self-concept has been associated with both general school achievement (Hattie, 1992, Valentine et al., 2004) and maths-specific achievement (Marsh, 1986).

*Maths interest* relates to people's intrinsic motivation to acquire new mathematical skills. The association between subject interest and achievement is complex. Stable interest in particular academic subjects has been found to predict achievement in those subjects but, indirectly, through the mediating effects of self-regulation (Lee, Lee, & Bong, 2014). In mathematical learning, maths interest has been studied to understand pupil motivation for engaging in mathematics activities (e.g. Wigfield & Cambria, 2010). Using a longitudinal design, Marsh, Köller, Trautwein, Lüdtke, and Baumert (2005) reported a positive correlation between mathematics interest and achievement that may have been mediated by a reciprocal relationship between interest and mathematics self-concept. This study also suggested that the mathematical component assessed may affect study outcomes as both mathematics self-concept and interest were more strongly associated with school grades (average r = 0.35) than with standardised mathematics tests (average r = 0.22).

There is evidence that individual differences in aspects of motivation emerge as early as primary school and that such factors predict future learning (Masters and Santrock, 1976, Mazzocco et al., 2012). A recent study found that motivation towards mathematics at age 11 accounted for variance in mathematics achievement at age 16 beyond that explained by cognitive ability (Murayama, Pekrun, Lichtenfeld, & vom Hofe, 2013). The researchers also found that motivation and learning strategies were associated with growth in achievement, whereas cognitive ability was associated with concurrent achievement but not growth (Murayama et al., 2013).

In addition to research reporting individual differences in motivation as early as primary school (Mazzocco et al., 2012), several studies have observed a general decline in academic motivation during early adolescence, which parallels a decline in academic achievement (Frenzel et al., 2010, Meece et al., 2006). Research investigating associations between self-perceived abilities and achievement could potentially help here by building an evidence-base for developing new systems aimed at maintaining pupils' interest in maths, and their self-perceived ability, at least to the extent that they reach the required standard for good educational and occupational choices at the end of compulsory schooling. Beyond that, intrapersonal factors such as maths self-efficacy, maths interest and academic self-concept appear related to decisions about pursuing advanced levels of mathematical training, or occupations with a mathematical component (Van den Broeck et al., 2005, Wang, 2012). Promoting interest and self-belief in students with the ability to pursue mathematics to a high level may be a relevant consideration.

### 1.4 Perceptions of the classroom environment

It is important to seek insight into the development of individual differences in intrapersonal factors which may vary across learning environments (Bandura, 2012). Several studies have found that *Classroom Environment*, or perceptions thereof, are related to both self-efficacy beliefs and maths achievement (Danielsen et al., 2010, Eccles and Roeser, 2011, Schunk, 1982, Schunk, 1984, Schunk and Hanson, 1985, Wentzel et al., 2010, Eshel and Kohavi, 2003). One study of the relationships between perceptions of the classroom environment, intrapersonal factors and maths achievement in 10 year-old children found that pupils who perceived their maths classrooms as caring, challenging and mastery oriented reported significantly higher levels of maths self-efficacy (Fast et al., 2010). In turn, having higher levels of maths self-efficacy was positively associated with maths performance. Interestingly, in this study self-reported classroom environment did not show any direct relationship with maths achievement. Conversely, the results of another study suggested a direct association between perceptions of the classroom environment, derived through observational measures and gains in test performance (Pianta et al., 2007). A further study, using self-reported perceptions of chemistry classrooms found an indirect relationship with both achievement and intrinsic motivation via achievement goals (Church, Elliot, & Gable, 2001). Inconsistencies in the literature may reflect different measures and definitions of classroom environment as well as complex inter-relationships between learning environments, intrapersonal factors and achievement. As academic motivation tends to decline with age, academic subject, age and developmental stage may also be important to perceived classroom environment. Two of the studies mentioned above were focused on pupils in middle childhood rather than adolescence and the third involved undergraduates studying chemistry (Church et al., 2001). It is possible that relationships between intrapersonal measures, environmental measures and achievement may differ by subject, and by age, and in the current study we focus specifically on mathematics at age 16.

### 1.5 Aims and hypotheses

Findings about inter-relationships between learning environments, achievement and intrapersonal factors have been somewhat heterogeneous. Some of this heterogeneity may have derived from how constructs are operationalized and which mathematical components have been assessed. The current study aimed to increase understanding of the relationship between mathematics as assessed by GCSE school achievement and tests, three intrapersonal factors, and a maths learning environment. It used data from a large representative sample of UK 16-year-old students, many of whom are at the end of their mathematics education (UK students are not required to pursue mathematics beyond GCSE). The sample is spread throughout the UK and was drawn from the full range of schools in the UK therefore controlling, to some extent, for school type.

The study aimed to explore whether, at this particular age and educational stage, a process of triadic reciprocal causation will be observed, and to look more closely at each of the relationships involved. In particular it is expected that:

1. Maths classroom environment will be associated with mathematics performance (both GCSE school achievement and maths tests) and three self-reported intrapersonal factors (maths self-efficacy, academic self-concept, maths interest).
2. The three intrapersonal variables will be associated with performance on both mathematics assessments.
3. Within the dynamic relationship between behaviour, personal factors and environment suggested by the triadic reciprocal causation model, intrapersonal factors will mediate the relationship between classroom environment and maths performance.

Academic self-concept was included, as well as maths-specific variables, in order to assess whether motivation towards mathematics is independent of general views of oneself as a learner.

## 2 Materials and method

### 2.1 Sample

Participants were drawn from the Twins' Early Development Study (TEDS). TEDS is an on-going longitudinal study of three cohorts of twins born in 1994, 1995 and 1996 (Oliver & Plomin, 2007). Families of twins were contacted through the Office for National Statistics and over 13,000 families were recruited across England and Wales. TEDS participants have been regularly tested throughout their lives. Data for this study was collected from 7448 twin pairs (male n = 3519) when they were 16 years old (mean = 16.48; SD = 0.27) using web-based tests and questionnaires. After excluding twins on the basis of medical problems or not having English as their first language, 6689 twin pairs with raw data remained (male n = 3150 pairs). The TEDS sample has been shown to be reasonably representative of the UK population of same-age adolescents and their parents (Oliver and Plomin, 2007, Haworth et al., 2013). Although participation in each wave of data collection is optional, active participants remain representative of the entire sample. As twins cannot be considered independent participants, analyses were conducted using a randomly chosen twin from each pair, and replicated in the co-twin sample.

### 2.2 Measures

The online battery (available at www.teds.ac.uk) containing mathematics tests and intrapersonal factors questionnaires, was administered to the first two cohorts of TEDS twins. GCSE grades were collected from all three cohorts using a self-reported postal questionnaire (details of sample size for each measure available in Table 1).

#### 2.2.1 Mathematics

Two aspects of mathematics were assessed using the online test battery.

*Understanding Number* assesses mathematical skills according to levels required at age 16 by the UK National Curriculum (NC). This test is made up of 18 items selected from National Foundation for Educational Research (NFER) booklets (Levels 1 to 8; nferNelson, 1994, nferNelson, 1999, nferNelson, 2001). NFER tests are developed and used for UK school assessments; *Understanding Number* questions aim to assess understanding of numerical processes and relationships between mathematical operations. A sample question is “Work out the value of x: 6x + 9 = 8x” and participants are asked to click on the correct solution from five choices. The second test, *Problem Verification Task*, is designed to assess mathematical fluency, the efficiency with which one can evaluate the veracity of an arithmetic solution. This test was adapted from a description in Murphy and Mazzocco (2008). It includes 48 arithmetic problems, each presented with a single solution. Participants are asked to judge whether the solution is correct. For instance, participants are presented with the following problem and solution: 28 ÷ 16 = 2. They are asked to respond as quickly as possible, within 10 s, by clicking keys corresponding to three responses: Right, Wrong and Don't Know. Both tests showed good reliability in the current sample (α = 0.90, n = 2153 for *Understanding Number*; and α = 0.85, n = 2238 for *Problem Verification Task*). The two measures were strongly correlated (r = 0.67) and were combined into a single maths accuracy score by averaging their standardised means.

Participants completed the tests online between June and September of 2010 and 2011, just after sitting their GCSE examinations. The on-line tests therefore represent roughly concurrent measures of achievement. *GCSE* grades were collected via post shortly after the official release of UK school examination results in August 2010, 2011 and 2012. Non-responders were followed up with telephone calls.

#### 2.2.2 Intrapersonal factors

Questionnaires assessing intrapersonal factors and perceived classroom environment were administered online at the same time as the mathematics web-tests.

*Maths Self-efficacy* was measured with 8 items drawn from PISA (Programme for International Student Assessment) student questionnaires (2000, 2003, 2006: www.pisa.oecd.org). Participants were asked how confident they would feel about undertaking a series of mathematically based tasks including calculating how much cheaper a TV would be after a 30% discount and understanding graphs presented in newspapers. Students were not asked to solve the problems, just to rate their confidence in being able to do so on a 4-point scale ranging from ‘Not At All Confident’ (0) to ‘Very Confident’ (3). The total score was computed as the mean of at least 4 items. This mean was multiplied by the total number of items in the test (8), generating a range of scores between 0 and 24 (in this sample α = 0.90, n = 2328).

*Maths Interest* used 3 items, also drawn from PISA questionnaires. The scale included items such as “I look forward to my mathematics lessons”. Ratings ranged from 1 (strongly disagree) to 4 (strongly agree). This short measure also showed good reliability (α = 0.93, n = 2382).

*Academic Self-concept* was measured using 10 items selected from a 20-item measure (Burden, 1998). Items included: “I′m good at doing tests” and “I find a lot of schoolwork difficult” which participants responded to on a 5 point scale ranging from ‘Very much like me’ to ‘Not like me at all’. This measure showed good reliability in our sample (α = 0.82, n = 2216).

*Perceived Classroom Environment* assesses perceptions of classroom climate during maths lessons and was measured using 17 items drawn from PISA and Midgley, Eccles, and Feldlaufer (1991). Participants were asked to think about their maths lessons in responding to items such as: “The teacher shows an interest in every student's learning”; and “There is noise and disorder”. Items were rated on a 4-point scale, with low scores corresponding to negative classroom environments (α = 0.88, n = 2405).

### 2.3 Analyses

A strength of our design is that it allowed for a built-in replication study, whereby we repeated the analyses using the sample of co-twins, therefore tables present results conducted on one half of the sample. Overall, highly similar patterns of results were observed in this matched sample. Only results significant in both samples are reported here as significant.

All analyses were conducted using variables standardised to a mean of 0.00 and a standard deviation (SD) of 1.00, corrected for age and with outliers (± 3 SDs) removed. Because previous studies have found sex differences in both maths achievement and motivational factors (e.g. Halpern et al., 2007, Spelke, 2005, Wigfield et al., 2002) we assessed our variables for sex effects using ANOVA in order to check whether it would be appropriate to combine males and females in a single sample.

GCSE Maths and web-test scores were highly correlated with each other (r = 0.74). Web-test items assess mathematical abilities required by UK NC levels, therefore mirroring the content of GCSEs. However, GCSEs may tap into broader cognitive abilities and factors associated with classroom experience that may not be captured by web-tests. We also hypothesised that they may be somewhat affected by different factors. For instance, the pressure of GCSE, a high-stakes assessment, may be pertinent to the classroom environment in a way that would not be the case for the on-line test. For these reasons, web-test scores and GCSE results were used as separate measures in all analyses.

In order to assess the strength of association between mathematics and perceived classroom environment/intrapersonal factors, GCSE grades and web-test scores were entered as dependent variables in two stepwise regressions. In both, classroom environment was entered into the first block. Intrapersonal factors were entered in the second block (forced entry) and web-test scores were entered in the third block when GCSE results were used as the dependent variable and GCSE results when web-test scores were the dependent variable.

Bandura's theory predicts that intrapersonal factors and perceptions of the classroom environment may be interrelated. We assessed the extent to which they have an influence on each other, beyond the influence exerted by test performance (behaviour), by using the three intrapersonal factors and perceived classroom environment as dependent variables. In separate models, each factor was used in turn as the dependent variable. Classroom environment was entered in the first block, remaining intrapersonal factors in the second, and both measures of test performance in the third. In a fourth model, classroom environment was used as the dependent variable, three intrapersonal factors were entered in the first block, and both measures of test performance in the second.

Mediation analyses were conducted to explore whether significant associations between classroom environment and maths achievement were mediated by intrapersonal factors. We conducted three simple mediation models wherein classroom environment was considered as a predictor of GCSE and each intrapersonal factor as a mediator. In a multiple-mediator model, all three intrapersonal measures were entered simultaneously. The same mediations were carried out using Maths Web-test as the dependent variable. Mediation analyses were conducted using PROCESS (Preacher and Hayes, 2008, Hayes, 2012). Bootstrapping with 5000 resampling was used in order to avoid sample bias. In all mediation analyses the bootstrap coefficients were almost identical to the data coefficient, indicating good sampling. We note that we used PROCESS, partly to enable multiple mediation analysis, but that our results were consistent with those generated using Sobel tests.

## 3 Results

### 3.1 ANOVAs

Descriptive statistics for raw and standardised data, regressed for age and cleared of outliers on all measures appear in Table 1. ANOVA analyses revealed no significant sex differences for perceived classroom environment. There were small but statistically significant sex differences in all other measures (e.g., boys scored higher in maths achievement GCSE and web-tests, academic self-concept, maths self-efficacy and subject interest). Although these results were statistically significant because of the large sample, effect sizes (partial eta-squared) were negligible-to-small, ranging from η^2^ = 0.00 for GCSE to η^2^ = 0.06 for maths self-efficacy. Levene's test indicated that the variances did not significantly differ in males and females for web-tests and GCSE grades. For the remaining variables, the assumption of equality of variance was not met, but the effect sizes of variance differences were small: ~ 1% for self-efficacy and interest and between zero and < 1% for the remaining variables. The results of these analyses justified conducting the investigation including males and females in the same sample.

### 3.2 Correlations

In line with Bandura's model, all measures correlated significantly with each other ranging from r = 0.24 for the association between maths web-test and classroom environment to r = 0.74 for the association between GCSE and web-tests (Table 2). Intrapersonal factors were consistently more strongly associated with mathematics (average correlation r = 0.52 with both GCSE and web-tests) than Classroom Environment was. The correlation of Classroom Environment with GCSE was slightly stronger (r = 0.28) than with web-tests (r = 0.24). Overall, Classroom Environment showed a stronger relationship with the three intrapersonal factors (average r = 0.35) than with achievement.

### 3.3 Multiple regression

#### 3.3.1 Predicting maths achievement

A stepwise regression incorporating only Classroom Environment significantly predicted performance in GCSE Maths, explaining 7% of the variance (Table 3, left hand-side). However, when intrapersonal variables were added, only these measures were significant predictors. Classroom Environment's contribution became non-significant, suggesting that most of its variance may be in common with one or more of the measured intrapersonal factors. In this model most of the variance was explained by Maths Self-efficacy (β = 0.52). When web-test score was included as a predictor in the third regression model, it became the strongest predictor (β = 0.54); Academic Self-concept remained significant, but Maths Interest was only a significant predictor in one of the two samples. Maths Self-efficacy was the second strongest independent predictor (β = 0.25) of GCSE performance. The full model predicted 61% of the variance in GCSE Maths.

A very similar pattern emerged when web-test scores were the dependent variable (Table 3, right panel). The full model accounted for 59% of the variance with GCSE Maths as the strongest predictor (β = 0.55), followed by Maths Self-efficacy (β = 0.22). It is notable that for mathematics web-tests Maths Interest was a significant predictor in the full model while it was non-significant for GCSE.

#### 3.3.2 Predicting self-efficacy, interest, self-concept and perceptions of the classroom environment

The regressions in Table 3 suggest intercorrelations among the maths predictors. Step-wise regression analyses were used to investigate statistical predictors of each of the intrapersonal factors and perceived classroom environment (Table 4).

When predicting maths self-efficacy, classroom environment was a significant predictor (β = 0.28) but only in the first step. When the other intrapersonal variables were added into the model, they offered significant prediction and Classroom Environment became non-significant. In this model, Academic Self-concept was the strongest predictor of Maths Self-efficacy (β = 0.42) and remained a strong predictor (β = 0.27) even when test performance was added into the mode. The full model explained 59% of the variance in self-efficacy (Table 4).

When predicting Maths Interest, Classroom Environment was a significant predictor on its own (β = 0.40) and remained significant when intrapersonal factors were added in the second step (β = 0.25) and test performance in the third (β = 0.24). These results suggest that perceived classroom environment contributes to interest in mathematics beyond mathematical skills and beyond general or maths-specific academic self-confidence. The strongest predictor of Maths Interest was Maths Self-efficacy (β = 0.33); interestingly, school achievement (GCSE) did not predict interest but web-test scores did, with small effects (β = 0.11). The full model explained 37% of the variance in maths interest (Table 4).

When predicting Academic Self-concept, Classroom Environment was again a significant predictor on its own (β = 0.30). It remained significant when the other intrapersonal factors were added in the second step and maths in the third. It can be observed that its association with Academic Self-concept weakened considerably after the inclusion of Maths Self-efficacy and Interest in the model. The strongest predictor of Academic Self-concept was Maths Self-efficacy (β = 0.42); both maths performance measures were independent but weak predictors of Academic Self-concept The full model explained 36% of the variance (Table 4).

When predicting perceptions of classroom environment, Maths Self-efficacy was not a significant predictor in any model while Maths Interest was the strongest predictor (β = 0.31). GCSE Maths significantly, but weakly, predicted perceived classroom environment (β = 0.07); the web-test scores did not. Overall, only a small portion of variance in classroom environment (18%) was explained in the full model and this was almost entirely accounted for by the three intrapersonal factors (ΔR^2^ from mathematics in the third step was non-significant). These analyses provide support for hypotheses 1 and 2; and point to reciprocal relationships between intrapersonal factors and achievement but we do not find evidence of a reciprocal relationship between maths performance and perceived classroom environment.

### 3.4 Mediation analyses

#### 3.4.1 Relationships between perceptions of classroom environment and achievement

It was notable in the regression analyses that mathematics performance was more strongly related to intrapersonal factors than to perceptions of the learning environment. However, the pattern of overlapping variances among variables made it difficult to interpret these associations. In order to make this clearer we conducted mediation analyses.

**Fig. 1:**
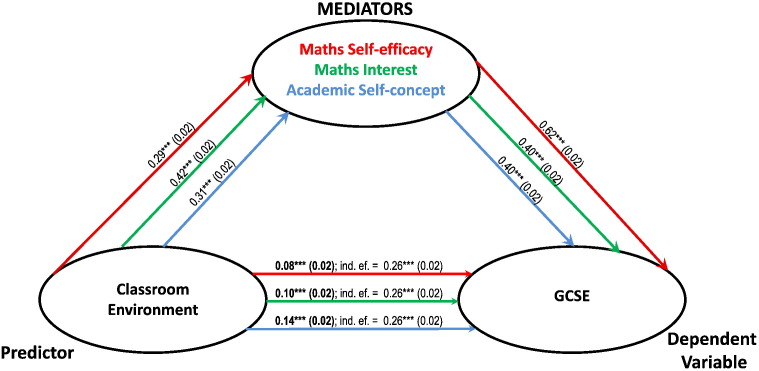
Summary of 3 distinct simple mediation models. In each model, Classroom Environment and GCSE are entered as predictor and dependent variable respectively, the 3 learning attitudes are individually entered as mediators. The paths are colour coded, ordered following the order of the mediators and report unstandardised beta coefficients (*** = p < 0.001) with their standard errors in brackets. The paths from the predictor to the dependent variable report the beta coefficient for the direct effects after mediation (in bold characters) and the indirect effects of the predictor before mediation (ind. ef.). For example, the effects of Classroom Environment on GCSE decrease from 0.26 (indirect effect) to 0.08 (direct effect) as result of the mediation of Maths Self-efficacy. As the direct effects are still significant after mediation, the mediation of Maths Self-efficacy is partial.

**Fig. 2:**
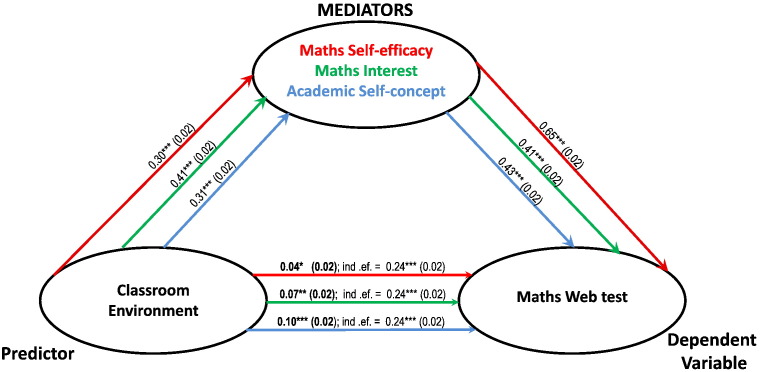
Summary of 3 distinct simple mediation models. In each model, Classroom Environment and Mathematics Web tests are entered as predictor and dependent variable respectively, the 3 learning attitudes are individually entered as mediators. The paths are colour coded, ordered following the order of the mediators and report unstandardised beta coefficients (*** = p < 0.001; ** = p < 0.01; * = p < 0.05) with standard errors in brackets. The paths from the predictor to the dependent variable report the beta coefficient for the direct effects in bold characters and the indirect effects of the predictor (ind. ef.).

Math Self-efficacy, Maths Interest and Academic Self-concept were entered one at a time as mediators between Classroom Environment and maths performance. Fig. 1 summarises the results of three simple mediation models with GCSE as the dependent variable; Fig. 2 summarises three simple mediations with web-test score as the dependent variable. It can be observed that each intrapersonal factor partially mediated the relationship between Classroom Environment and both types of test performance. Math Self-efficacy was the strongest mediator, reducing the correlation between classroom environment and GCSE from 0.26 to 0.08 (Fig. 1); and between Classroom Environment and Maths web-test score from 0.24 to 0.04 (Fig. 2). Using Maths Interest as mediator reduced the correlation between Classroom Environment and GCSE from 0.26 to 0.10 (Fig. 1); and between Classroom Environment and web-test score from 0.24 to 0.04 (Fig. 2). Academic Self-concept was the weakest of the mediators and reduced the correlation between classroom environment and GCSE from 0.26 to 0.14 (Fig. 1); and between Classroom Environment and web-test score from 0.24 to 0.10 (Fig. 2).

**Fig. 3:**
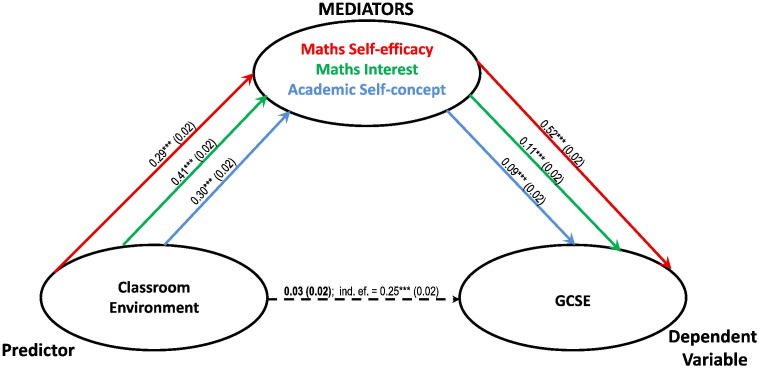
Multiple mediators model. Classroom Environment and GCSE are entered as predictor and dependent variable respectively; the 3 learning attitudes are simultaneously entered as mediators. The paths are colour coded, ordered following the order of the mediators and report unstandardised beta coefficients (*** = p < 0.001) and their standard errors in brackets. The dashed arrow between the predictor and the dependent variable represents the direct effects of Classroom Environment (noted in bold characters) on GCSE. This is what is left of its influence after partialling out the cumulative influences of the 3 mediators. Together, Maths Self-efficacy, Maths Interest and Academic Self- concepts totally mediate the relationship between Classroom Environment and GCSE, as after mediation the correlation decrease from 0.25 (p < 0.001) to 0.03, non-significant.

**Fig. 4:**
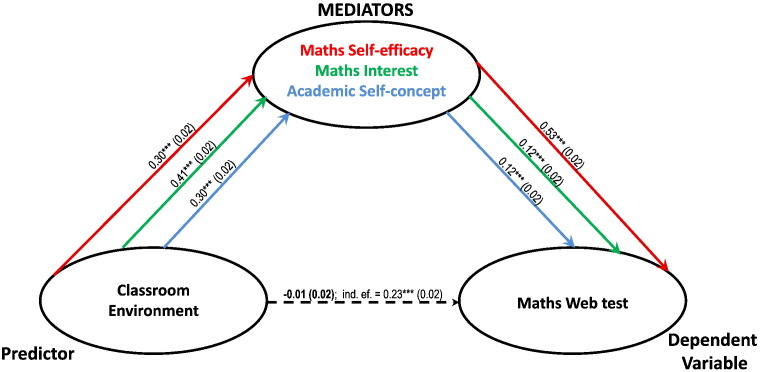
Multiple mediators model. Classroom Environment and Maths Web tests are entered as predictor and dependent variable respectively; the 3 learning attitudes are simultaneously entered as mediators. The mediators colour coded paths report unstandardised beta coefficients (*** = p < 0.001) with standard errors in brackets. The direct effects of Classroom Environment on GCSE are non-significant (dashed arrow). Together, Maths Self-efficacy, Maths Interest and Academic Self- concepts totally mediate the relationship between Classroom Environment and Maths Web tests, as after mediation the correlation decrease from 0.23 (p < 0.001) to non-significant.

From the regressions in Table 3 it can be seen that Classroom Environment does not significantly predict mathematics performance once intrapersonal factors are taken into account. In fact, when intrapersonal factors were entered simultaneously in two multiple-mediators models, one where Classroom Environment predicted GCSE (Fig. 3) and another where it predicted maths web-test (Fig. 4) they totally mediated the relationship between Classroom Environment and mathematics. The single mediation models however, allow understanding of the influence of each of the intrapersonal factors in the relationship between environment and achievement.

## 4 Discussion

### 4.1 Findings and implications

Associations between mathematics achievement and classroom environment, maths self-efficacy, subject interest and academic self-concept were investigated using Bandura's triadic reciprocal causation model as a framework (Bandura, 1986). In line with hypothesis 1, maths test performance (behaviour) was predicted by intrapersonal and environmental factors. It is important to note that mathematics performance was more strongly correlated with the three intrapersonal factors than with perceived classroom environment (hypothesis 2). In fact when intrapersonal factors were included in the regression analyses, the association between perceived classroom environment and mathematics became non-significant. Conversely, after controlling for mathematics, classroom environment contributed with unique variance to two of the intrapersonal factors (maths interest and academic self-concept. This was suggestive of mediation (hypothesis 3). Indeed, individually, intrapersonal factors partially mediated the relationship between classroom environment and maths performance and, together, they fully mediated it.

Overall, these study results are in line with previous research reporting stronger correlations between intrapersonal factors and maths performance than between perceived classroom environment and performance. The current study adds to this literature by analysing data from a sample of 16-year-olds rather than younger children (Fast et al., 2010) or chemistry undergraduates (Church et al., 2001). It appears that perceived maths environment does not exceed intrapersonal factors as a correlate of maths performance at this developmental stage, and this appears to be true across the environments provided by different types of school as the students in our sample attended the full range of UK schools.

In line with the triadic reciprocal causation model, our measures showed intercorrelations. However, the relationship between classroom environment and mathematics almost disappeared when all the mediators were taken into account. The fact that the relationship between perceived classroom environment and achievement was mediated by intrapersonal factors does not imply that perceptions of the learning environment don't matter. It needs to be noted that among the intrapersonal factors measured in the current study, Maths Self-efficacy was the strongest predictor of achievement. Perceived classroom environment was not predictive of Maths Self-efficacy, but it was an independent predictor of Interest and Self-concept. As these latter were predictors of Maths Self-efficacy we could hypothesise that improving perceptions of the classroom environment may influence mathematics achievement, via self-efficacy, by boosting factors such as interest and self-belief. This is important at a time when policymakers in the UK are showing a clear interest in non-cognitive traits, sometimes referred to as ‘character’, in schools. Such findings can be taken into account when considering ways of improving learning attitudes as an indirect means of enhancing achievement.

In line with the literature, our study found an indirect association of classroom environment and mathematics. However, the mediation analyses sheds light on some discrepancies in the literature (e.g. Pianta et al., 2007), as these analyses highlight that direct effects may still be detected as an artefact of the variables assessed in a study.

The lack of a significant correlation between maths self-efficacy and classroom environment in the presence of maths interest and academic self-concept may suggest that self-efficacy differs across domains and that maths-specific self-efficacy operates differently from general academic self-concept, therefore, identifying environmental mechanisms for enhancing maths self-efficacy remains a research priority.

### 4.2 Limitations and future directions

It could be considered a limitation that all participants were members of a twin-pair. However, twins have been shown to differ very little from non-twin siblings beyond early childhood, and therefore this is unlikely to have influenced our findings (e.g. Deary, Spinath, & Bates, 2006).

A more important limitation of our design is that we have captured a still-frame at the age of 16 of a dynamic process that may have started before formal education even began. As correlational designs cannot address cause and effect, it remains necessary to explore the observed relationships using a longitudinal approach. This is especially relevant in light of evidence that individual differences in intrapersonal factors earlier in childhood, and the cumulative effects of learning environments over time, may influence maths achievement (Pianta et al., 2007) and that differences in the emergence of motivation towards maths are observed as early as age seven (Mazzocco et al., 2012) Further, there is evidence of reciprocal relationships between self-perceived maths abilities and maths performance (e.g. Luo, Kovas, Haworth, & Plomin, 2011) as well as directional relationships. Prior achievement in mathematics appears to drive motivation towards the subject (Garon-Carrier et al., 2016, Spinath et al., 2006) and intrinsic motivation (enjoyment in learning without external rewards), significantly declines between the age 9 and 17 years (Gottfried, Marcoulides, Gottfried, Oliver, & Guerin, 2007). Our results suggest that at age 16 the association of Maths Self-efficacy and Academic Self-concept with achievement is indeed reciprocal. Interest however, seemed less strongly linked to concurrent achievement. At age 16, perceptions of classroom environments contributed with independent variance to interest in mathematics which, in turn, contributed independently to maths achievement. A longitudinal approach to the current findings therefore represents an important avenue for future research. This may help us to better understand how to use classroom environments to raise maths interest and, perhaps, to counter at earlier ages the chain reaction leading to the well-documented decline in maths interest and achievement.

Although our study has enhanced understanding of the dynamic underpinning associations among the measured domains, our design did not allow us to address the true nature of such relationships. It will be important to study observed associations among achievement, perceived classroom environment and intrapersonal factors using a genetically informative design. Recent evidence suggests that the relationship between general academic self-evaluation and achievement may be mediated genetically (Krapohl et al., 2014). However, this research did not focus directly on mathematics and did not explore the role of maths interest. We therefore plan, as Part 2 of this research programme, to carry out a twin analysis of intrapersonal factors, perceptions of the maths learning environment and mathematical outcomes at age 16. The relationship between subject interest and classroom environment appears to hold particularly strong educational interest. Our critical next step will be to conduct a genetically sensitive study of this relationship. Being able to ascertain how much of the variance among these domains can be accounted for by genes and how much by common and individual specific environmental factors, will allow us to gain greater understanding of how to develop new ways of promoting interest and positive self-perceptions, and therefore perhaps achievement. Kovas et al. (2015) found that 60% of the variance in motivation is explained by non-shared environmental (NSE) factors. A genetically-informed study will allow us to explore whether perceptions of the classroom can explain NSE variance in motivation, either directly or indirectly via interest.

We consider the current and proposed future research to be an important endeavour given the gatekeeping nature of GCSE Maths and the strong relationship between maths self-efficacy and maths achievement (and to a lesser extent, maths interest, academic self-concept and achievement). How can we use what we know about maths education to help more young people cross the Grade C threshold? This proposed research may also offer new insight into initiatives designed to encourage participation in mathematics beyond GCSE. The current findings suggest that improving perceptions of the classroom environment may boost interest and self-belief, and thereby achievement. It is possible that strategies designed to improve mathematical outcomes should focus on students' perceptions of their learning environments as well as objective test-performance.

## Figures and Tables

**Table 1:** Means, standard deviations and ANOVA results by sex.

Measures	Means and standard deviations for raw data including outliers		Means and standard deviations for standardised data						ANOVA-effects of sex	
	All		All		Females		Males		Sex	
	M (n)	SD	M (n)	SD	M (n)	SD	M (n)	SD	p	η^2^
1 GCSE	8.93 (n = 5274)	9.00	0.04 (n = 5218)	0.96	0.01 (n = 2772)	0.97	0.07 (n = 2446)	0.95	0.02	0.00
2 Maths Web test composite	–	–	0.02 (n = 2303)	1.00	− 0.13 (n = 1350)	0.99	0.24 (n = 953)	0.98	0.00	0.03
3 Academic Self-concept	3.56 (n = 2215)	3.60	0.01 (n = 2210)	0.99	− 0.12 (n = 1301)	1.01	0.20 (n = 909)	0.92	0.00	0.03
4 Maths Self-Efficacy	17.67 (n = 2403)	19.00	0.02 (n = 2385)	0.96	− 0.17 (n = 1391)	0.99	0.30 (n = 994)	0.85	0.00	0.06
5 Maths Interest	2.54 (n = 2404)	2.67	0.01 (n = 2404)	1.00	− 0.09 (n = 1405)	1.03	0.14 (n = 999)	0.94	0.00	0.01
6 Classroom Environment	32.34 (n = 2391)	33.00	0.02 (n = 2381)	0.98	− 0.01 (n = 1388)	1.02	0.06 (n = 993)	0.93	0.07	0.00

M = mean; SD = standard deviation; n = sample size; p = p-value of the effects of sex on variables; η^2^ = eta-squared. Means and standard deviations reported on the standardised scores refer to variables correct for age and cleared of outliers scores (± 3 standard deviations). Mean and standard deviation for the Maths web composite is not provided as this is derived averaging the standardised means of the two mathematics web tests. The table presents results of analyses conducted on half of the sample constituted by one randomly selected twin in each pair. Sample size is larger for GCSE scores because data was collected on the 3 cohorts, while for all the other measures, the web data was collected only on 2 cohorts.

**Table 2:** Correlations.

			1	2	3	4	5	6
1	GCSE	n	5218					
		r	0.74⁎⁎					
2	Maths Web-test	n	2035	2303				
		r	0.46⁎⁎	0.46⁎⁎				
3	Academic Self-concept	n	1961	2200	2210			
		r	0.64⁎⁎	0.64⁎⁎	0.58⁎⁎			
4	Maths Self-efficacy	n	2098	2277	2191	2385		
		r	0.47⁎⁎	0.45⁎⁎	0.41⁎⁎	0.54⁎⁎		
5	Maths interest	n	2110	2291	2202	2384	2404	
		r	0.28⁎⁎	0.24⁎⁎	0.31⁎⁎	0.31⁎⁎	0.42⁎⁎	
6	Classroom Environment	n	2094	2269	2182	2362	2380	2381

n = sample size; r = Pearson's correlation. Pearson's correlations are conducted on half of the sample constituted by one randomly selected twin in each pair. Variables are corrected for age and cleared of outliers (± 3 standard deviations).

⁎⁎ p < 0.01 level (2-tailed).

**Table 3:** Regression - Mathematics predicted.

Predictor measures	GCSE Maths Predicted			Maths Web-test Predicted		
	Standardised coefficients			Standardised coefficients		
	β	t	η^2^	β	t	η^2^
*1st step: Perceived learning environment*						
Classroom Environment	0.26	11.90⁎⁎		0.23	10.47⁎⁎	
	F(1,1924) = 141.61; p = 0.000; R^2^ = 0.07			F(1,1924) = 109.65; p = 0.000; R^2^ = 0.05		

*2nd step: Intrapersonal factors added*						
Classroom Environment	0.03	1.58 (♦)		0.00	-0.17	
**Maths Self-efficacy**	0.52	22.33⁎⁎		0.50	21.41⁎⁎	
**Maths Interest**	0.12	5.62⁎⁎		0.13	6.21⁎⁎	
**Academic Self-concept**	0.10	4.62⁎⁎		0.10	4.86⁎⁎	
	F(4,1921) = 377.51; p = 0.000; R^2^ = 0.44, Fchange(3,1921) = 424.94, ΔR^2^ = 0.37; p = 0.000			F(4,1921) = 353.73; p = 0.000; R^2^ = 0.42, Fchange(3,1921) = 411.68, ΔR^2^ = 0.37; p = 0.000		

*3rd step: Maths added*						
Classroom Environment	0.03	1.99 (♦)	0.08	-0.02	-1.22	0.06
Maths Self-efficacy	0.25	11.42⁎⁎	0.41	0.22	9.80⁎⁎	0.41
Maths Interest	0.05	2.63⁎ (♦)	0.22	0.07	3.72⁎⁎	0.20
Academic Self-concept	0.04	2.34⁎	0.21	0.05	2.77⁎	0.21
**Maths Web-test**	0.54	28.52⁎⁎	0.55	–	–	
**GCSE Maths**	–	–		0.55	28.52⁎⁎	0.55
	F(5,1920) = 592.38; p = 0.000; R^2^ = 0.61, Fchange(1,1920) = 818.33, ΔR^2^ = 0.17; p = 0.000			F(5,1920) = 565.31; p = 0.000; R^2^ = 0.59, Fchange(1,1920) = 813.33, ΔR^2^ = 0.17; p = 0.000		

The table reports results of analyses conducted on half of the sample constituted by one randomly selected twin in each pair. The analyses replicated in the sample of the co-twins are very similar. The symbol (♦) indicates when significance is discrepant in the two samples; R^2^ reports the adjusted value of the percentage of variance explained in the model. ΔR^2^ represents the change in R^2^ in the new step, resulting by the addition of the variables. Only results significant in both samples are reported significant in the overall analyses. The variables added in the model are noted with bold characters.

⁎⁎ p < 0.001.

⁎ p ≤ 0.05.

**Table 4:** Step-wise regressions; intrapersonal factors and perceived learning environment predicted.

Predictor measures	Maths Self-efficacy Predicted			Maths Interest Predicted			Academic Self-Concept Predicted			Classroom Environment Predicted		
	β	t	η^2^	β	t	η^2^	β	t	η^2^	β	t	η^2^
*1st step: Perceived learning environment*												
Classroom Environment	0.30	13.87⁎⁎		0.40	18.90⁎⁎		0.30	13.63⁎⁎		–	–	
	F(1,1924) = 192.23; p = 0.000; R^2^ = 0.09			F(1,1924) = 357.34; p = 0.000; R^2^ = 0.16			F(1,1924) = 185.78; p = 0.000; R^2^ = 0.09					

*2nd step: Intrapersonal factors added*												
Classroom Environment	0.03	1.63		0.25	12.63⁎⁎		0.12	5.80⁎⁎		–	–	
**Maths Self-efficacy**	–	–		0.43	18.98⁎⁎		0.50	22.80⁎⁎		0.46	0.16	
**Maths Interest**	0.37	18.98⁎⁎		–	–		0.07	3.18⁎		0.31	12.63⁎⁎	
**Academic Self-concept**	0.42	22.80⁎⁎		0.07	3.18⁎		–	–		0.15	5.80⁎⁎	
	F(3,1922) = 532.55; p = 0.000; R^2^ = 0.45, Fchange(1,1922) = 638.98, ΔR^2^ = 0.36; p = 0.000			F(3,1922) = 359.98; p = 0.000; R^2^ = 0.36, Fchange(2,1922) = 304.87, ΔR^2^ = 0.20; p = 0.000			F(3,1922) = 349.53; p = 0.000; R^2^ = 0.35, Fchange(2,1922) = 393.50, ΔR^2^ = 0.27; p = 0.000			F(3,1922) = 141.95; p = 0.000; R^2^ = 0.18		

*3rd step: Maths added*												
Classroom Environment	0.02	0.99	0.10	0.24	12.34⁎⁎	0.17	0.11	5.66⁎	0.09	–	–	–
Maths Self-efficacy	–	–		0.33	12.12⁎⁎	0.30	0.42	15.74⁎⁎	0.34	0.03	0.99	0.10
Maths Interest	0.22	12.13⁎⁎	0.30	–	–		0.05	2.29⁎	0.17	0.31	12.34⁎⁎	0.17
Academic Self-concept	0.27	15.74⁎⁎	0.34	0.05	2.29⁎	0.17	–	–		0.15	5.66⁎⁎	0.10
**GCSE Maths**	0.26	11.42⁎⁎	0.41	0.08	2.64⁎ (♦)	0.22	0.07	2.34⁎	0.21	0.07	1.98⁎	0.08
**Maths Web-test**	0.22	9.80⁎⁎	0.41	0.11	3.72⁎⁎	0.20	0.08	2.77⁎	0.21	− 0.04	− 1.22 (♦)	0.06
	F(5,1920) = 546.12; p = 0.000; R^2^ = 0.59, Fchange(2,1920) = 309.79, ΔR^2^ = 0.13; p = 0.000			F(5,1920) = 230.04; p = 0.000; R^2^ = 0.37, Fchange(2,1920) = 22.85, ΔR^2^ = 0.02; p = 0.000			F(5,1920) = 218.49; p = 0.000; R^2^ = 0.36, Fchange(2,1920) = 14.54, ΔR^2^ = 0.01; p = 0.000			F(5,1920) = 86.05; p = 0.000; R^2^ = 0.18, Fchange(2,1920) = 1.99, ΔR^2^ = 0.002; p = 0.14		

The table reports results of analyses conducted on half of the sample constituted by one randomly selected twin in each pair. The analyses replicated in the sample of the co-twins are very similar. The symbol (♦) indicates when significance is discrepant in the two samples; R^2^ reports the adjusted value of the percentage of variance explained in the model. ΔR^2^ represents the change in R^2^ in the new step, resulting by the addition of the variables. Only results significant in both samples are considered significant in the overall analyses. The variables added in the model are noted with bold characters.

⁎⁎ p < 0.001.

⁎ p < 0.05.

## References

[bb0005] Bandura A. (1977). Self-efficacy: Toward a unifying theory of behavioral change. Psychological Review.

[bb0010] Bandura A. (1986). Social foundations of thought and action: A social cognitive theory.

[bb0015] Bandura A. (2012). On the functional properties of Self-Efficacy Revisited. Journal of Management.

[bb0025] Burden (1998). Assessing children's perceptions of themselves as learners and problem solvers. School Psychology International.

[bb0030] Bynner J., Parsons S. (2005). Does numeracy matter more?.

[bb9000] Church M.A., Elliot A.J., Gable S.L. (2001). Perceptions of classroom environment, achievement goals, and achievement outcomes. Journal of Educational Psychology.

[bb0040] Danielsen A.G., Wiium N., Wilhelmsen B.U., Wold B. (2010). Perceived support provided by teachers and classmates and students' self-reported academic initiative. Journal of School Psychology.

[bb0045] Deary I.J., Spinath F.M., Bates T.C. (2006). Genetics of intelligence. European Journal of Human Genetics.

[bb0050] Deary I.J., Strand S., Smith P., Fernandes C. (2007). Intelligence and educational achievement. Intelligence.

[bb0060] Dulaney A., Vasilyeva M., O'Dwyer L. (2015). Individual differences in cognitive resources and elementary school mathematics achievement: Examining the roles of storage and attention. Learning and Individual Differences.

[bb0065] Eccles J.S., Roeser R.W. (2011). Schools as developmental contexts during adolescence. Journal of Research on Adolescence.

[bb0070] Eccles J.S., Wigfield A. (2002). Motivational beliefs, values, and goals. Annual Review of Psychology.

[bb0080] Elliot A.J., Dweck C.S. (2005). Handbook of competence and motivation.

[bb0085] Eshel Y., Kohavi R. (2003). Perceived classroom control, self-regulated learning strategies, and academic achievement. Educational Psychology.

[bb0090] Fast L.A., Lewis J.L., Bryant M.J., Bocian K.A., Cardullo R.A., Rettig M., Hammond K.A. (2010). Does math self-efficacy mediate the effect of the perceived classroom environment on standardized math test performance?. Journal of Educational Psychology.

[bb0095] Frenzel A.C., Goetz T., Pekrun R., Watt H.M.G. (2010). Development of mathematics interest in adolescence: Influences of gender, family and school context. Journal of Research on Adolescence.

[bb0100] Garon-Carrier G., Boivin M., Guay F., Kovas Y., Dionne G., Lemelin J.P., Tremblay R.E. (2016). Intrinsic motivation and achievement in mathematics in elementary school: A longitudinal investigation of their association. Child Development.

[bb0105] Geary D.C. (2011). Cognitive predictors of achievement growth in mathematics: A 5-year longitudinal study. Developmental Psychology.

[bb0110] Gottfried A.E., Marcoulides G.A., Gottfried A.W., Oliver P.H., Guerin D.W. (2007). Multivariate latent change modeling of developmental decline in academic intrinsic math motivation and achievement: Childhood through adolescence. International Journal of Behavioral Development.

[bb0115] Hackett G., Betz N.E. (1989). An exploration of the mathematics self-efficacy/mathematics performance correspondence. Journal for Research in Mathematics Education.

[bb0120] Halpern D.F., Benbow C.P., Geary D.C., Gur R.C., Hyde J.S., Gernsbacher M.A. (2007). The science of sex differences in science and mathematics. Psychological Science in the Public Interest.

[bb0125] Hattie J. (1992). Self-concept.

[bb0135] Haworth C.M.A., Davis O.S.P., Plomin R. (2013). Twins Early Development Study (TEDS): A genetically sensitive investigation of cognitive and behavioral development from childhood to young adulthood. Twin Research and Human Genetics.

[bb0140] Hayes A.F. (2012). PROCESS: A versatile computational tool for observed variable mediation, moderation, and conditional process modeling [white paper]. http://www.afhayes.com/public/process2012.pdf.

[bb0150] Hoffman B., Schraw G. (2009). The influence of self-efficacy and working memory capacity on problem-solving efficiency. Learning and Individual Differences.

[bb0155] Joint Council for Qualifications (2014). http://www.jcq.org.uk.

[bb0170] Kovas Y., Garon-Carrier G., Boivin M., Petrill S.A., Plomin R., Malykh S.B., Brendgen M. (2015). Why children differ in motivation to learn: Insights from over 13,000 twins from 6 countries. Personality and Individual Differences.

[bb0175] Krapohl E., Rimfeld K., Shakeshaft N.G., Trzaskowski M., McMillan A., Pingault J.B., Plomin R. (2014). The high heritability of educational achievement reflects many genetically influenced traits, not just intelligence. Proceedings of the National Academy of Sciences.

[bb0180] Lee W., Lee M.J., Bong M. (2014). Testing interest and self-efficacy as predictors of academic self-regulation and achievement. Contemporary Educational Psychology.

[bb0185] Luo Y.L., Kovas Y., Haworth C., Plomin R. (2011). The etiology of mathematical self-evaluation and mathematics achievement: Understanding the relationship using a cross-lagged twin study from ages 9 to 12. Learning and Individual Differences.

[bb0190] Marsh H.W. (1986). Verbal and math self-concepts: An internal/external frame of reference model. American Educational Research Journal.

[bb0195] Marsh H.W., Craven R.G. (2006). Reciprocal effects of self-concept and performance from a multidimensional perspective: Beyond seductive pleasure and unidimensional perspectives. Perspectives on Psychological Science.

[bb0200] Marsh H.W., Köller O., Trautwein U., Lüdtke O., Baumert J. (2005). Academic self-concept, interest, grades, and standardized test scores: Reciprocal effects models of causal ordering. Child Development.

[bb0205] Masters J.C., Santrock J.W. (1976). Studies in the self-regulation of behavior: Effects of contingent cognitive and affective events. Developmental Psychology.

[bb0210] Mazzocco M.M., Hanich L.B., Noeder M.M. (2012). Primary school age students' spontaneous comments about math reveal emerging dispositions linked to later mathematics achievement. Child Development Research.

[bb0215] Meece J.L., Anderman E.M., Anderman L.H. (2006). Classroom goal structure, student motivation, and academic achievement. Annual Review of Psychology.

[bb0220] Midgley C., Eccles J.S., Feldlaufer H., Fraser B.J., Walberg H.J. (1991). Classroom environment and the transition to junior high school. Educational environments: Evaluation, antecedents and consequences.

[bb0225] Murayama K., Pekrun R., Lichetnfeld S., vom Hofe R. (2013). Predicting long-term growth in students' mathematics achievement: the unique contributions of motivation and cognitive strategies. Child Development.

[bb0230] Murphy M.M., Mazzocco M.M.M. (2008). Mathematics learning disabilities in girls with fragile x or turner syndrome during late elementary school. Journal of Learning Disabilities.

[bb1000] nferNelson (1994). Mathematics 5–14 series.

[bb2000] nferNelson (1999). Mathematics 5–14 series.

[bb3000] nferNelson (2001). Mathematics 5–14 series.

[bb0235] OECD (2010). The high cost of low educational performance: The long-run economic impact of improving educational outcomes.

[bb0240] Oliver B.R., Plomin R. (2007). Twins' Early Development Study (TEDS): A multivariate, longitudinal genetic investigation of language, cognition and behavior problems from childhood through adolescence. Twin Research and Human Genetics.

[bb0245] Pajares F. (1996). Self-efficacy beliefs in academic settings. Review of Educational Research.

[bb0250] Pianta R.C., Belsky J., Houts R., Morrison F. (2007). Opportunities to learn in America's elementary classrooms. Science (New York, N.Y.).

[bb0255] Pietsch J., Walker R., Chapman E. (2003). The relationship among self-concept, self-efficacy, and performance in mathematics during secondary school. Journal of Educational Psychology.

[bb0260] PISA (OECD Programme for International Student Assessment: 2000, 2003, 2006). www.pisa.oecd.org.

[bb0265] Preacher K.J., Hayes A.F. (2008). Asymptotic and resampling strategies for assessing and comparing indirect effects in multiple mediator models. Behavior Research Methods.

[bb0275] Robbins S.B., Lauver K., Le H., Davis D., Langley R., Carlstrom A. (2004). Do psychosocial and study skill factors predict college outcomes? A meta-analysis. Psychological Bulletin.

[bb0280] Rohde T.E., Thompson L.A. (2007). Predicting academic achievement with cognitive ability. Intelligence.

[bb0285] Schunk D.H. (1982). Effects of effort attributional feedback on children's perceived self-efficacy and achievement. Journal of Educational Psychology.

[bb0290] Schunk D.H. (1984). Enhancing self-efficacy and achievement through rewards and goals: Motivational and informational effects. The Journal of Educational Research.

[bb0295] Schunk D.H., Hanson A.R. (1985). Peer models: Influence on children's self-efficacy and achievement. Journal of Educational Psychology.

[bb0305] Spelke E.S. (2005). Sex differences in intrinsic aptitude for mathematics and science. A critical review. American Psychologist.

[bb0310] Spinath B., Spinath F.M., Harlaar N., Plomin R. (2006). Predicting school achievement from general cognitive ability, self-perceived ability, and intrinsic value. Intelligence.

[bb0315] Swanson H.L., Sachse-Lee C. (2001). Mathematical problem solving and working memory in children with learning disabilities: Both executive and phonological processes are important. Journal of Experimental Child Psychology.

[bb0320] Taub G.E., Keith T.Z., Floyd R.G., McGrew K.S. (2008). Effects of general and broad cognitive abilities on mathematics achievement. School Psychology Quarterly.

[bb0325] Tosto M.G., Hanscombe K.B., Haworth C., Davis O.S., Petrill S.A., Dale P.S., Kovas Y. (2014). Why do spatial abilities predict mathematical performance?. Developmental Science.

[bb0335] Valentine J.C., DuBois D.L., Cooper H. (2004). The relation between self-beliefs and academic achievement: A meta-analytic review. Educational Psychologist.

[bb0340] Van den Broeck A., Opdenakker M.C., Van Damme J. (2005). The effects of student characteristics on mathematics achievement in Flemish TIMSS 1999 data. Educational Research and Evaluation.

[bb0345] Vukovic R.K., Lesaux N.K. (2013). The relationship between linguistic skills and arithmetic knowledge. Learning and Individual Differences.

[bb0350] Wang M.T. (2012). Educational and career interests in math: A longitudinal examination of the links between classroom environment, motivational beliefs, and interests. Developmental Psychology.

[bb0355] Wentzel K.R., Battle A., Russell S.L., Looney L.B. (2010). Social supports from teachers and peers as predictors of academic and social motivation. Contemporary Educational Psychology.

[bb0360] Wigfield A., Cambria J. (2010). Students' achievement values, goal orientations, and interest: Definitions, development, and relations to achievement outcomes. Developmental Review.

[bb0365] Wigfield A., Battle A., Keller L.B., Eccles J.S., McGillicuddy-De Lisi A. (2002). Sex differences in motivation, self-concept, career aspiration, and career choice: Implications for cognitive development. The development of sex differences in cognition.

[bb0370] Williams T., Williams K. (2010). Self-efficacy and performance in mathematics: Reciprocal determinism in 33 nations. Journal of Educational Psychology.

[bb0375] Young M.R. (2005). The motivational effects of the classroom environment in facilitating self-regulated learning. Journal of Marketing Education.

